# Supplementary material for: Patterns of Intron Gain and Loss in Fungi
Source: PLoS Biol. 2004 Nov 30;2(12):e422. doi: 10.1371/journal.pbio.0020422 (PMC532390; doi:10.1371/journal.pbio.0020422)
Supplement: Table S1 — Also available at http://genes.mit.edu/NielsenEtAl/. (4.3 MB ZIP). [file pbio.0020422.st001.zip › NielsenEtAl/html/244.html]

AN0473.1.NCU06305.1.MG06644.1.FG04827.1


```
 CLUSTAL W (1.82) Multiple Sequence Alignments - Introns Inserted


Sequence 1: MG06644.1	497 aa
Sequence 2: FG04827.1	507 aa
Sequence 3: AN0473.1	507 aa
Sequence 4: NCU06305.1	516 aa
Alignment Length: 520 aa
Number Identitical Residues: 289 aa
Alignment Score (without introns) 13332


MG06644.1 	MSTPE---AK-EMGHVDPGANCLSGG---------HGEGLVMDPSRRAVVEKSMKRK0LD
NCU06305.1	MARHG--GRDSLSGIEKPAVEASENYTHNEQPNTGNLNVRAMDPEERIRIEKSLLRK~LD
FG04827.1 	MADQK---PDDHMATAKTSVESQSRGP--------TAIALAMDPQHRAAVEKSMKRK~LD
AN0473.1  	MADDTKLGSDALQ-LEKPTVETME----------GGYPASRMSPARRAEVEKSMKRK~LD
          	*:   . . .      .. .:  .          .      *.* .*  :***: ** **

MG06644.1 	LRCSLFVLIYIM~NYLD0RNNIAAARLGTLEKDLNLEYNQYATCLSILYVGYILMQV1PS
NCU06305.1	ARCSYFVVIYIL2NYLD~RNNIGAARLKGLQEDLKLTDTQYATCLSILYVGYILMQI~PS
FG04827.1 	TRCALFVLIYIM~NYLD~RNNMAAARLKGLQEDLNLSYNQYATCLSILYVGYILMQV~PS
AN0473.1  	ARCSLFVLIYIM~NYLD~RNNMAAARLKGLQEDLNMNYDEYATCLSILYVGYILMQV~PS
          	 **: **:***: **** ***:.****  *::**::   :****************: **

MG06644.1 	NIFINRIESPSLYISCAMLLWGLISTLSGNAQTFAHMVVIRFFLGFVEAAFLPGALLLLS
NCU06305.1	NMFINRIPRPSLYISAVMAIWGLISTLTGLAKGFGGMVAIRFLLGFVEAAFLPGALLILS
FG04827.1 	NMFINRIQRPSLYISAAMLLWGLISTLSGSVHNFAGMVSIRFFLGFVEAAFLPGALLILS
AN0473.1  	NILINRIQRPSLYISLVMTLWGLISTLSGVVHNFSGMVAIRFFLGFVEAAFLPGALMILS
          	*::****  ****** .* :*******:* .: *. ** ***:*************::**

MG06644.1 	KWYTRKELALRNAILFCGNLISNAFSALVGAGVLSNMHG2VLGHSAWRWLFWT1------
NCU06305.1	KWYTRRELTKRNAILFCGNLISNAFSSLVGAGVLSNMDG~VLGHRAWRWLFYI~EGAATM
FG04827.1 	KWYTRSELTKRNAILFCGNLISNAFSALVGAGVLSNMQG~VLGHAAWRWLFWI~EGAITM
AN0473.1  	KWYTRRELTTRNAILFCGNLISNAFSALIGAGVLSNMQG~VLGHAAWRWLFWI1EGAATM
          	***** **: ****************:*:********.* **** ******:  ..: : 

MG06644.1 	---ISAMFILPDLPHNSKGFTEEERHVAQLRMTEDVGEADVDSAEQGMFDGLIMTLKDFK
NCU06305.1	FVAVMAAFILPDLPTNSRGFTEEELLVAQLRMTEDVGEADSDSADMGTFDGLIMVVKDWK
FG04827.1 	GVAVSAAFILPDLPHNSRGFTEEERQVAQLRMVEDVGEADEDSREESAFYGLKLAVKDVK
AN0473.1  	TIAIAAAIILPDLPSNTRGFTQEELEVAQLRMTEDVGEADVDSEDSGPWDGFFMAIKDVK
          	  :: * :****** *::***:**  ******.******* ** : . : *: :.:** *

MG06644.1 	VYL2MCIIFTCYVIGLSFNAFF~PSLTGTLGFDYVPTLLMSSPPWAFSCIVSLINAWHSD
NCU06305.1	VYL~MMLTFTAYTTGLSFNAFF0PTLTGTLGLAYVPTLLLSAPPWVFACIFSLINAWHSD
FG04827.1 	IYI~MMLTFTAYVVGLSFNAFF0PSLTQTLGFGYVPTLLMSAPPWVFSCIVSVINAWHSD
AN0473.1  	MYI~MMLTLTAYVVGLSFNAFF0PSLTETLGFAYVPTLLMSAPPWVFSCLFSLCVAWSSD
          	:*: * : :*.*. ******** *:** ***: ******:*:***.*:*:.*:  ** **

MG06644.1 	KTQEK0FWHIVGPVFVGIIGFVISMATDNTAGRYVALFLQASSYAG~FIVFYSWISSSFP
NCU06305.1	RKQEK~FGHIVGPICMGLVGFVISMATLNKAARYVALFLQAGSYAG~FIVFYSWISSSFP
FG04827.1 	KTQEK0FWHIVGPICVGTIGFIISMATEAVAARYLALFLQASSYAG~FIVFYSWISSSFP
AN0473.1  	RYQEK0FWHIVIPMLMGLVGFIICMSTLQTAARYVALFLQAGSYAG2FIVFYSWISTSFP
          	: *** * *** *: :* :**:*.*:*   *.**:******.**** *********:***

MG06644.1 	RPPAKRAVALALVNAFSQLGNVAGSYVWNLKANGYRLSYAIVLVMFVITAVGCWVFRLIL
NCU06305.1	RPPAKRAVALAMINAFSQLGNVAGSYVWDLKENGYRKSYGIVTAMFGVTVVGCWVIRMVL
FG04827.1 	RPPAKRAVAIAMINAFSQLGNVAGSYVWDLKENGYRKSYGIVLSMFGVTVLGCYIFRLVL
AN0473.1  	RPPAKRAVAIAMVNAFSQLGNVAGSYVWDLKENGYRKSYGIVTAMFGVAIVGCLIFKLIL
          	*********:*::***************:** **** **.**  ** :: :** :::::*

MG06644.1 	QNLNKKLDEGEA-AWETQADVAQQTAAVEKLGDSDDQRLERMRRQFRYMV
NCU06305.1	VDQNKKLAHGEESAWEVHDDVANQTARVEGLKSEGAD--LKPQIGFRYLV
FG04827.1 	IDLNKKLERGEATAWETRQDVAAHTAAVEVMDSPDE--ALRMKRDFRYLI
AN0473.1  	EALNKKLAEAEAVEVSSGAGAAQQSGMHGGEGDMDES--LRMHKGFRYLV
          	   **** ..*    .   ..* ::.      . . .   : :  ***::
```
